# Advancing liquid biopsies in pediatric brain tumors: unlocking the clinical utility of circulating cell-free small RNAs

**DOI:** 10.1007/s44307-026-00144-5

**Published:** 2026-11-13

**Authors:** Chiara Dossena, Matilde Oriani, Veronica Biassoni, Olga Nigro, Elisabetta Schiavello, Linda Gregorio, Roberta Mortarini, Alessandra Molla, Claudia Vegetti, Maura Massimino, Loris De Cecco

**Affiliations:** 1 Integrated Biology of Rare Tumors, Department of Experimental Oncology, Fondazione IRCCS Istituto Nazionale Dei Tumori, AmadeoLab, Via Amadeo N. 42, Milan, 20133 Italy; 2 Pediatric Oncology Unit, Fondazione IRCCS Istituto Nazionale Dei Tumori, Milan, Italy; 3 Human Tumors Immunobiology Unit, Department of Experimental Oncology, Fondazione IRCCS Istituto Nazionale Dei Tumori, Milan, Italy

**Keywords:** Liquid biopsies, Circulating small RNA, Pediatric brain tumors, MiRNA, PiRNA, tsRNA CircRNA

## Abstract

Tissue specimens remain the gold standard for diagnosis and molecular characterization of cancer, but their acquisition is invasive and often difficult to repeat, particularly in pediatric neuro-oncology. Liquid biopsies offer a minimally invasive approach for obtaining tumor-related information from accessible biofluids. Blood, including plasma and serum, is the most widely investigated matrix, while cerebrospinal fluid (CSF) is biologically closer to central nervous system (CNS) tumors and may provide a higher tumor-derived signal. Molecular profiling through liquid biopsies therefore has considerable potential to support precision medicine, longitudinal disease assessment, and treatment monitoring in children with brain tumors. Among circulating regulatory RNAs, canonical small RNAs include microRNAs (miRNAs), PIWI-interacting RNAs (piRNAs), and tRNA-derived small RNAs (tsRNAs), whereas circular RNAs (circRNAs) represent a structurally and size-distinct RNA class that can also be detected in biofluids. Current pediatric clinical evidence is strongest for circulating miRNAs, with studies supporting potential diagnostic, prognostic, disease-monitoring, and treatment-response assessment applications across several pediatric CNS tumor entities. In contrast, circulating piRNAs and tsRNAs remain largely supported by adult, mixed-age, or preclinical glioma studies and require dedicated validation in pediatric cohorts. Adult-derived findings remain valuable as hypothesis-generating translational evidence when pediatric data are unavailable, but should not be considered directly transferable without independent pediatric validation. Interpretation of circulating RNA signatures also requires consideration of their biological origin. Circulating RNAs may derive directly from tumor cells, but can also reflect immune and inflammatory responses, blood-cell or platelet contributions, treatment effects, and blood–brain or blood-tumor barrier permeability. Their detection in peripheral blood therefore cannot be attributed simply to small molecular size, and circulating profiles may not faithfully reproduce the tumor RNA landscape. Clinical translation further depends on rigorous control of pre-analytical and analytical variables, including biofluid selection, hemolysis, collection and processing procedures, storage, RNA isolation, normalization, and platform choice. Although small-RNA sequencing, RT-qPCR, and digital PCR provide complementary opportunities for discovery and validation, most circulating RNA biomarkers in pediatric brain tumors remain investigational. Prospective, multicenter validation in molecularly defined pediatric cohorts, standardized workflows, demonstration of analytical and clinical performance, and independent clinical utility assessment will be essential before these biomarkers can be integrated into routine patient management.

**Supplementary Information:**

The online version contains supplementary material available at https://doi.org/10.1007/s44307-026-00144-5.

## Introduction

Biomarkers are of outmost importance for diagnosis, prognostication and treatment prediction in neuro-oncology (Eroles and Rico 2023). Although tissue specimens are the gold standard in oncology for diagnosis and for revealing the biological alterations of cancer cells, tissue sampling requires invasive surgical procedures, with limited repeatability and potential morbidity. In pediatric patients in particular, surgical access to brain tumors can be more challenging because of the location of the tumor and the limited operative space, potentially increasing the technical complexity and morbidity associated with neurosurgical procedures. Therefore, minimally invasive biomarkers in liquid biopsies, such as circulating small RNAs, could be particularly valuable in this population, as they provide a means of obtaining molecular information related to the tumor without invasive intervention. In addition, tissue sampling often provides a single spatiotemporal snapshot of the tumor, whereas liquid biopsies allow the course of the disease to be followed during treatment and, eventually, at progression. In the last decade, the need for alternative methods to diagnose central nervous system (CNS) tumors and monitor their behavior has, emerged particularly in the pediatric population. Their rarity and molecular heterogeneity also limit the availability of large cohorts, independent validation datasets, and prospective clinical studies. Consequently, when evidence specific to pediatric patients is lacking, studies of biologically related adult tumors can provide valuable translational and hypothesis-generating information, including candidate biomarkers, mechanisms, and analytical strategies. However, pediatric brain tumors cannot be regarded as age-restricted counterparts of adult CNS tumors, because they differ in molecular drivers, epigenetic landscapes, developmental context, and clinical behavior (Jones et al. 2012; Abedalthagafi et al. 2021; Louis et al. 2021). In addition, adult-derived findings require independent pediatric validation. Importantly, pediatric evidence is expanding, particularly for circulating miRNAs, although most studies remain small, exploratory, and require prospective multicenter validation.

Liquid biopsies (LBs) are an attractive source of material due to their non-invasive and easily accessible nature, offering a promising tool for determining the genomic profile of cancer, monitoring treatment responses, and identifying the onset of resistance (Madlener and Gojo 2020). Among the various biological fluids, blood (plasma and serum) remains the primary medium for detecting cancer biomarkers, although other samples, such as urine and cerebrospinal fluid (CSF), are also suitable (Connal et al. 2023).

Despite significant advancements in LB technologies, most research has focused on adult-type tumors, leading to the development of tailored panels that often overlook the unique characteristics of pediatric tumors (Louis et al. 2021). These tumors, while less common, exhibit a wide range of histologic, genomic, and clinical subtypes. Notably, CNS tumors are the leading cause of cancer-related mortality in children and represent the second most prevalent type of pediatric cancer after leukemia (Subramanian and Ahmad 2024).

Molecular profiling of tumors through LBs holds great promise for advancing precision medicine and facilitating the development of targeted therapies. Biofluids used for liquid biopsy contain circulating tumor cells, circulating tumor DNA, proteins, conventional circulating RNAs and several classes of regulatory non-coding RNAs. Among the latter, canonical small RNAs include miRNAs, piRNAs and tRNA-derived fragments, whereas circRNAs represent a structurally and size-distinct RNA class that may also be detected in extracellular fluids and extracellular vesicles (Madlener and Gojo 2020). These components have been shown to cross the blood–brain barrier (BBB), presenting an opportunity for the minimally invasive monitoring of brain tumors (Mori et al. 2019). Circulating tumor DNA (ctDNA) has been extensively studied, tools such as next-generation sequencing (NGS) panels have already entered clinical practice. Both ctDNA and circulating small RNAs can enter extracellular biofluids through passive release associated with cell death, including apoptosis and necrosis, as well as through mechanisms involving extracellular vesicles and other extracellular carriers. Although apoptosis and necrosis are considered major contributors to ctDNA release, active DNA export has also been reported. Similarly, circulating small RNAs may originate from dying cells or undergo regulated secretion by viable cells, including selective packaging into extracellular vesicles. Therefore, differences between ctDNA and circulating small RNAs should not be interpreted as a strict distinction between passive and active release, but rather in terms of their release mechanisms, extracellular stability, molecular carriers, and potential biological information (Gahlawat et al. 2023; Shurtleff et al. 2016). In this context, circulating small RNAs may provide biological information complementary to ctDNA, potentially reflecting not only tumor-associated molecular alterations but also changes in cellular activity, stress responses, and interactions with the tumor microenvironment. However, their circulating levels may also be influenced by non-tumor sources and systemic physiological or pathological conditions (Stejskal et al. 2023). Compared with their adult counterparts, pediatric cancers are generally characterized by a lower mutational burden and fewer recurrent mutations, but show greater epigenetic dysregulation and more chromosomal aberrations (Jones et al. 2019). Epigenomic alterations include not only methylation but also small non-coding RNAs, such as miRNAs or piRNAs.

As such, small RNAs represent a promising area of research with the potential to provide insight into CNS tumor biology and disease biomarkers (Tang et al. 2020). Circulating miRNAs are attractive candidate biomarkers because they can be released in protected extracellular forms, including extracellular vesicles (EVs), ribonucleoprotein complexes, and lipoprotein-associated particles, which increase their stability in biofluids. CNS-derived extracellular RNAs can become detectable in peripheral blood; however, their passage should not be attributed simply to their small molecular size. Rather, transport from the CNS to the systemic circulation is likely to depend on carrier-associated trafficking, active endothelial transcytosis, changes in BBB and blood–tumor barrier (BTB) permeability, and CSF clearance pathways. The relative contribution of these mechanisms remains incompletely defined and may vary according to tumor type, anatomical site, disease stage, and BBB integrity (Arvanitis et al. 2020; Ramos-Zaldívar et al. 2022; Morad et al. 2019).

A recent comprehensive review (Janssen et al. 2024) reported the relevance of liquid-biopsy applications in themost common non-CNS pediatric solid tumors, highlighting their potential as early disease biomarkers and in several other clinical applications.

Given the impact of pediatricCNS tumors on cancer-related mortality in children, there is a clear need to investigate the molecular profiles of these tumors and the presence of specific biomarkers in body fluids (Tang et al. 2020).

**Fig. 1:**
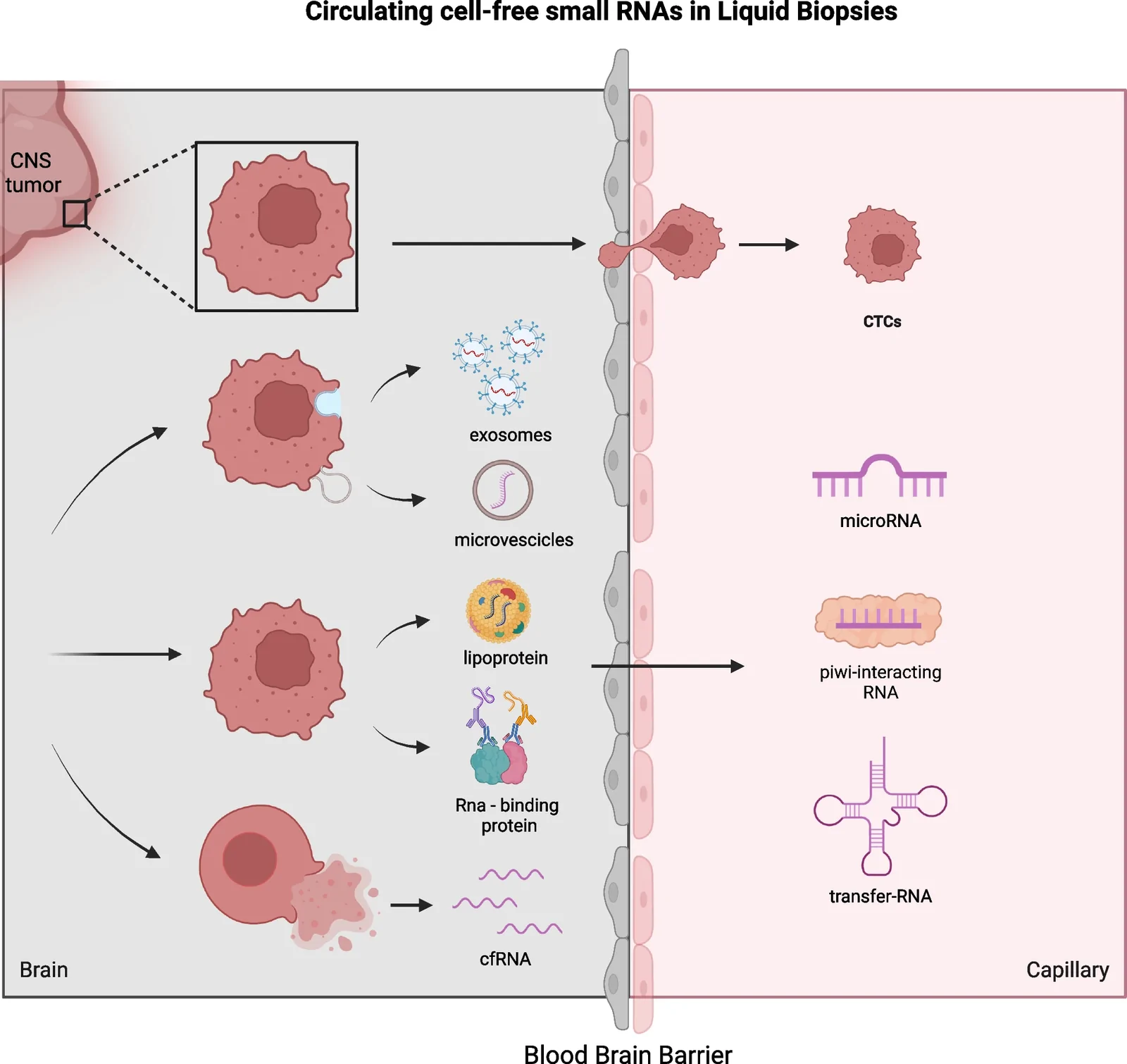
Tumor-derived circulating RNA biomarkers released by pediatric CNS tumors can reach biofluids and be investigated through liquid biopsy. These include canonical small RNAs, such as miRNAs, piRNAs and tRNA-derived fragments, as well as structurally distinct circRNAs. These RNA species may circulate in extracellular vesicles or in association with other extracellular carriers. Circulating tumor cells should be considered a separate cellular liquid-biopsy analyte rather than an RNA species

Given the limited number of reviews addressing the application of circulating small RNAs in pediatric brain tumors (D’Antonio et al. 2025; Katsushima et al. 2023; Shen et al. 2022), this review aims to fill that gap by providing a comprehensive summary of existing research on circulating small RNA biomarkers associated with pediatric brain tumors, while also discussing the technical challenges of developing liquid-biopsy biomarker assays for pediatric patients and potential future advances (Fig. 1). Unlike existing reviews primarily focused on adult brain tumors or individual RNA classes, this review specifically addresses pediatric CNS tumors by integrating evidence on circulating miRNAs, piRNAs, and tRNA-derived small RNAs (tsRNAs) within a unified framework that distinguishes pediatric clinical evidence from adult-derived and preclinical findings, while also examining the analytical, clinical validation, and regulatory requirements for their potential implementation in precision oncology.

## Pathways of small RNA secretion

Three main pathways of small RNA secretion have been identified: (1) passive release through cellular damage caused by apoptosis or necrosis; (2) active secretion facilitated by extracellular vesicles (EVs), including exosomes and microvesicles (MVs); (3) active secretion that involves RNA-binding proteins and lipoprotein complexes (Zirak et al. 2024). EVs, including both small EVs and microvesicles, contain lipids, proteins, mRNAs and small RNAs. However, their relative abundance in plasma or serum strongly depends on the pre-analytical and separation methods used, and lipoprotein particles in the EV size range outnumber EVs in blood by at least one order of magnitude and co-purify with them, which may confound EV-associated small RNA profiles (Sódar et al. 2016). EVs are secreted by a wide range of cell types and are critical mediators of intercellular communication, transferring information from one cell to another. Pediatric brain tumor cells, for instance, release exosomes whose miRNA repertoire differs from that of normal cells (Tűzesi et al. 2017). Tumor cells are particularly active in exosome production, which can also affect distant sites by contributing to the formation of pre-metastatic niches (Schiera et al. 2024).

In addition to small EVs and microvesicles, apoptotic bodies, i.e., large membrane-bound vesicles released during apoptotic cell disassembly and typically measuring approximately 1–5 µm in diameter (Kerr et al. 1972; Atkin-Smith et al. 2015; Caruso and Poon 2018), are also found in biological fluids and may carry RNA. However, their RNA cargo is dominated by ribosomal RNA, whereas small RNAs are relatively enriched in small-EV fractions (Crescitelli et al. 2013). Because the size ranges of EV subtypes overlap (approximately 30–150 nm for exosomes, 100–1000 nm for microvesicles/ectosomes, and from sub-micrometer apoptotic microvesicles to micrometer-sized apoptotic bodies), size alone cannot assign an EV to a specific biogenetic subtype. Accordingly, MISEV2023 recommends operational terms such as ‘small EVs’ and ‘large EVs’ unless the subcellular origin is demonstrated (Kalra et al. 2016; Welsh et al. 2024). Moreover, small RNAs can be released independently of vesicles: a large fraction of cell-free miRNAs in plasma is not vesicle-associated but bound to Ago2-containing ribonucleoprotein complexes (Kalluri and LeBleu 2020; Arroyo et al. 2011; Turchinovich et al. 2011). Other non-vesicular extracellular particles, including exomeres (~35 nm) and supermeres, as well as lipoproteins, may also carry extracellular RNA and can be co-isolated with EVs (Zhang et al. 2018; Zhang et al. 2021; Sódar et al. 2016). Ago2 is part of the RNA-induced silencing complex (RISC), where it contributes to miRNA-mediated gene silencing (Sheu-Gruttadauria et al. 2019; Fath et al. 2022). Recent research has demonstrated that these non-vesicular miRNAs are actively secreted from neurons. Interestingly, their release occurs from a specialized cellular region, the distal axon (Antoniou et al. 2023). Additionally, high-density lipoproteins (HDLs) have been identified as another component involved in intercellular communication and the transport of small RNAs. Indeed, the discovery that HDL can transport functional miRNAs has paved the way for additional profiling studies, which have revealed the presence of various other classes of small RNAs associated with HDL. These HDL-sRNA complexes typically contain RNA molecules ranging from 10 to 60 nucleotides in length (Vickers and Michell 2021). Notably, other categories of small RNAs derived from the host have been identified. Furthermore, small RNAs originating from nonhost sources, such as bacteria, fungi, and viruses linked to the microbiome, diet, and environment, have also been detected on circulating lipoproteins (Allen et al. 2018). However, the origin of the lipoprotein-associated fraction of cell-free miRNAs remains an open question (Castleberry et al. 2023).

It is uncertain whether the small RNAs found in LB from pediatric patients with CNS tumors accurately mirror the expression profiles of the tumors themselves. Few studies have investigated this by analyzing paired serum and tumor tissue samples to determine whether there is a correlation or mismatch in miRNA expression. Most miRNA changes detected in the serum of these patients, which are strongly linked to the presence of a brain tumor, do not correspond to the miRNA expression levels in the tumor tissue. This indicates that these peripheral miRNA alterations are likely derived from non-tumor tissues (Bookland et al. 2020). In short, ct-miRNAs in serum or plasma correlate with the presence of a specific CNS tumor, but they do not strictly mirror the tumor miRNome itself, partly because of the presence.of the BBB. At least four non-mutually exclusive processes can shape a circulating small RNA signature in a patient with a CNS tumor. First, passive or active release directly from tumor cells is thought to dominate for classical tumor-associated miRNAs such as miR-21, and it plausibly explains why several circulating signatures fall after surgical resection or effective treatment (Taylor 2015). Second, the host systemic and local immune response contributes substantially: a large fraction of the miRNAs most often reported as cancer biomarkers are also highly expressed by myeloid and lymphoid blood cells, so that their plasma levels can track circulating white-cell counts as much as tumor burden (Pritchard et al. 2012). Third, non-specific systemic inflammation and pre-analytical handling, including hemolysis and platelet activation occurring during blood drawing and processing, release erythrocyte- and platelet-derived miRNAs (e.g., miR-451 a, miR-486) that can be mistaken for disease-related changes if not controlled for. Fourth, treatment itself reshapes the circulating small RNA pool: corticosteroids, routinely used to control peritumoral edema in patients with brain tumors, alter circulating and tumor-infiltrating myeloid and lymphoid populations (Swildens et al. 2022), while radiotherapy induces distinct changes in circulating and exosomal miRNA profiles that are independent of any change in tumor burden. Distinguishing the relative contribution of these different sources is critical for the correct biological and clinical interpretation of circulating small RNA signatures. A biomarker whose signal is driven mainly by tumor-cell release, such as the circulating miR-371~373/miR-302–367 cluster in intracranial germ-cell tumors, which is essentially absent from non-tumor tissue, is a stronger candidate for diagnostic use and for tracking minimal residual disease (Murray et al. 2020; Schönberger et al. 2023). In contrast, a biomarker that is substantially confounded by immune or inflammatory activity may still hold value for monitoring treatment response or disease-associated systemic effects, but should not be interpreted as a direct readout of tumor burden, and is less suited to diagnostic applications where specificity for the tumor itself is required. Future studies in pediatric CNS tumors should therefore, where feasible, pair circulating small RNA profiling with parallel assessment of blood-cell counts, hemolysis indices, corticosteroid exposure, and treatment timing, and should explicitly benchmark candidate biomarkers against paired tumor tissue and against non-tumor control conditions (e.g., post-surgical resection, non-neoplastic inflammatory states) before assigning them a specific diagnostic or monitoring role. The BBB constitutes an additional biological filter between CNS-derived extracellular RNA and the systemic circulation. In particular, freely circulating RNA molecules are unlikely to cross an intact BBB simply as a consequence of their small size. In contrast, EV-associated RNAs may undergo active transcellular transport across brain endothelial cells. Experimental models have demonstrated EV uptake through endocytic pathways followed by endothelial transcytosis, with clathrin-dependent endocytosis, macropinocytosis, and endosomal recycling among the proposed mechanisms. Tumor-derived EVs have also been shown experimentally to cross an apparently intact BBB through transcytosis. In brain tumors, an additional mechanism is provided by disruption of the neurovascular unit and formation of a heterogeneous BTB, which can increase paracellular or transcellular leakage; importantly, however, tumor-associated barrier disruption is spatially heterogeneous and cannot be assumed to occur uniformly throughout a CNS tumor. CSF-mediated clearance and lymphatic drainage may provide additional routes by which CNS-derived extracellular material reaches the systemic circulation. Conversely, direct evidence that non-vesicular Ago2- or lipoprotein-associated small RNAs cross the BBB remains limited. Thus, different extracellular small RNA carriers should not be assumed to have equivalent CNS-to-blood transport properties (Arvanitis et al. 2020; Morad et al. 2019; Ramos-Zaldívar et al. 2022).

## Biological and clinical roles of circulating small RNAs and related circular RNAs

### Translational evidence for circulating RNA biomarkers in pediatric brain tumors

The discovery that many small RNAs are detectable in cell-free biofluids and display distinct expression patterns linked to various disease states highlights their potential as biomarkers for diagnosis, prognosis, and treatment monitoring. Under pathological conditions, these small RNAs can serve as chemical messengers in cell-to-cell communication, being transported to other cells via EVs. Table 1 summarizes the key findings in the field of pediatric CNS tumors.

**Table 1:** Clinical and preclinical studies of small RNAs relevant to liquid-biopsy applications in pediatric brain tumors. Pediatric studies are presented first, followed by adult or mixed-age brain-tumor studies included as translational context. For each study, the table reports the investigated RNA candidates or signatures, proposed clinical or biological role, cohort or model size, sample type, study population, and experimental setting. Studies based exclusively on tumor datasets, cell lines, or cell-derived extracellular vesicles provide biological or mechanistic support but do not constitute direct evidence of circulating biomarkers. Most pediatric candidates remain at an exploratory, single-cohort stage and require independent, preferably multicenter and prospective, validation before clinical implementation. The validation status reported for each study distinguishes internal analytical or statistical validation, evaluation in a separate patient cohort, independent supporting evidence from subsequent studies, and experimental functional validation. Where independent clinical validation was not reported, this is explicitly indicated. Validation in adult or mixed-age populations should not be interpreted as pediatric-specific clinical validation. Studies available only as conference abstracts are identified accordingly. ^a Described as exosomes by the original authors; terminology updated to EVs/small EVs in accordance with MISEV2023, as the endosomal origin of the isolated vesicles was not demonstrated

Small RNA	Role	Reference	N° patients/samples	Sample type	Study population	Model	Validation
miR-21, miR-15 b, miR-23 a, miR-146 b	Associated with tumor nodular size and treatment response in juvenile pilocytic astrocytoma	Bookland et al. 2018	7 JPA patients, 5 controls	Serum	Pediatric	Clinical	Independent validation not reported
miR-130 a, miR-145, miR-335	Differentially expressed in pediatric astrocytoma; candidate diagnostic biomarkers	López-Aguilar et al. 2017	9 patients with astrocytic tumors, 3 pediatric controls	Serum	Pediatric	Clinical	Independent validation not reported
Circulating miRNA profiles	Evaluation of serum/tumor concordance across pediatric brain-tumor histologies; circulating miRNA alterations did not consistently mirror the tumor miRNome	Bookland et al. 2020	10 brain tumors (3 JPA, 4 medulloblastoma, 3 ependymoma), 5 pediatric controls	Serum and matched tumor tissue	Pediatric	Clinical	Exploratory study; no independent validation
miR-4714-3p, miR-6090, miR-4505, miR-551 b-5p, miR-6089, miR-3960, miR-936, miR-1207-5p, miR-202-3p, miR-3676-5p, miR-4634, miR-4539, miR-4299	Prognostic signature for disease progression in DIPG	Iannó et al. 2022	47 DIPG patients	Serum	Pediatric/AYA	Clinical	Separate validation cohort; single-center study
miR-182-5p, miR-10 b-5p, miR-106 b-3p, miR-25-3p, miR-21-5p	Candidate diagnostic biomarkers for pediatric gliomas and glioma subgroups	Rogachevsky et al. 2026	68 pediatric glioma patients, 12 healthy controls	Plasma	Pediatric	Clinical	Independent validation not reported
miR-371 ~ 373 and miR-302/367 clusters	Overexpressed in malignant intracranial GCTs; candidate diagnostic biomarkers	Murray et al. 2020; Schönberger et al. 2023	3 intracranial malignant GCTs, 1 Langerhans cell histiocytosis comparator; 8 intracranial malignant GCTs, 12 healthy controls	Serum and/or CSF	Pediatric/AYA	Clinical	Findings supported by a subsequent independent study; distinct cohorts and methods
miR-517 a	Candidate diagnostic and disease-monitoring biomarker for ETMR; blood levels associated with tumor burden	Madlener et al. 2023	5 ETMR patients	Serum/plasma; CSF also evaluated	Pediatric	Clinical	Exploratory study; independent validation not reported
Cell-free miRNA profiles	miRNA patterns associated with tumor histology, grade, dissemination, and survival	Coyle et al. 2022 (conference abstract)	54 pediatric brain-tumor patients; CSF available for 33 and plasma for 53	CSF and plasma	Pediatric	Clinical	Conference abstract; independent validation not reported
miR-101-3p, miR-423-5p	Differentially expressed in medulloblastoma plasma EVs^a; functional tumor-suppressive activity	Xue et al. 2022	35 medulloblastoma patients, 17 healthy controls	Plasma EVs	Pediatric	Clinical + in vitro/in vivo validation	Functional validation in experimental models; independent clinical validation not reported
EV small-RNA/ncRNA profile	Broad CSF EV RNA profiling during therapy; profiles differed at diagnosis and partially normalized after induction chemotherapy, supporting longitudinal monitoring	Bell et al. 2022 (conference abstract; Head Start 4)	27 medulloblastoma patients, 11 non-tumor controls	Serial CSF EVs^a	Pediatric	Prospective clinical/translational	Conference abstract; independent validation not reported
CSF miRNA profile (87 differentially expressed miRNAs)	Candidate biomarkers for measurable residual disease and early recurrence in ependymoma	Chapman et al. 2024, BIOMECA/SIOP Ependymoma II	12 pediatric ependymomas, 8 controls	CSF	Pediatric	Clinical, exploratory	Exploratory study; independent validation not reported
miR-1290, miR-1246	Associated with stemness and invasive properties of pediatric glioma stem cells	Tűzesi et al. 2017	Pediatric glioma stem-cell models	Cell-culture-derived small EVs^a	Pediatric-derived	In vitro	Preclinical study; no clinical validation
miR-10 b, miR-21	Increased in CSF of patients with glioblastoma and brain metastases	Teplyuk et al. 2012	Adult CNS tumor cohort	CSF	Adult	Clinical	Ongoing (clinical trial NCT01849952)
miR-3180-3p, miR-5739	Candidate diagnostic biomarkers for glioblastoma	Bustos et al. 2022	32 primary GBM, 13 recurrent GBM, 73 healthy controls	Plasma	Adult	Clinical	Internal validation
17-cfRNA signature including piR-28347	Glioma-associated CSF cfRNA signature	Huang et al. 2025	18 gliomas, 46 meningiomas, 21 tumor-free CNS diseases	CSF and plasma	Mixed-age, predominantly adult	Clinical	Internal validation
4-cfRNA signature including piR-2325	Meningioma-associated cfRNA signature	Huang et al. 2025	18 gliomas, 46 meningiomas, 21 tumor-free CNS diseases	CSF and plasma	Mixed-age, predominantly adult	Clinical	Internal validation
piR_016658, piR_016659, piR_020829, piR_020490	Differentially represented in glioma EVs; candidate markers distinguishing GBM from lower-grade astrocytoma in the discovery material	Hallal et al. 2020	12 GBM, 5 grade II–III astrocytomas in discovery cohort; additional serum-EV validation cohort	CUSA-derived EVs and serum EVs	Adult	Clinical/translational	Separate serum-EV validation cohort reported; adult population
tRF-19-R118LOJX, tRF-19-6SM83OJX	Associated with LGG/GBM biology; suppress proliferation, migration, and vasculogenic mimicry in glioma models	Wei et al. 2023	TCGA datasets and glioma cell lines	Tumor datasets/cell lines	Non-pediatric	In silico + in vitro	In silico analysis and in vitro functional validation; no clinical liquid-biopsy validation
tRF-21-RZ81JJ6R0, tRF-20-RZ81JJ6R	Plasma expression associated with poor postsurgical outcome	Huang et al. 2025	18 gliomas within a mixed CNS-tumor cohort	Plasma	Mixed-age, predominantly adult	Clinical	Internal validation
tRF-18-07QSNHD2, tRF-21-RNLIK8980	Downregulated in CSF from glioma compared with meningioma	Huang et al. 2025	18 gliomas within a mixed CNS-tumor cohort	CSF	Mixed-age, predominantly adult	Clinical	Internal validation
tRF-32-FZ9KR959NRZZP	Candidate diagnostic and prognostic glioma biomarker	Huang et al. 2025	18 gliomas	CSF	Mixed-age, predominantly adult	Clinical	Internal validation

*Abbreviations*: *AYA* adolescents and young adults, *cfRNA* cell-free RNA, *CNS* central nervous system, *CSF* cerebrospinal fluid, *DIPG* diffuse intrinsic pontine glioma, *ETMR* embryonal tumor with multilayered rosettes, *EVs* extracellular vesicles, *GBM* glioblastoma, *GCTs* germ-cell tumors, *JPA* juvenile pilocytic astrocytoma, *LGG* low-grade glioma, *miRNA* microRNA, *ncRNA* non-coding RNA, *piRNA* PIWI-interacting RNA, *tRF* transfer RNA-derived fragment

Supplementary Table 1 highlights the biological functions, tumor associations, supporting evidence, and potential translational relevance of circulating small RNAs in this field. Because direct pediatric evidence is uneven across the different RNA classes, the studies discussed below should be interpreted according to both the age population investigated and the experimental setting. This distinction is particularly important in neuro-oncology because pediatric brain tumors are not simply age-restricted counterparts of adult disease. The current WHO classification separates adult-type and pediatric-type diffuse gliomas on the basis of distinct molecular drivers, epigenetic landscapes, developmental contexts, and clinical behavior, while pediatric cancers more generally differ from their adult counterparts in cellular origin, genetic complexity, driver alterations, and underlying mutational processes (Jones et al. 2019; Louis et al. 2021). Accordingly, an RNA biomarker identified in adult glioblastoma cannot be assumed to retain the same diagnostic, prognostic, or biological significance in pediatric gliomas without independent validation. In the studies reviewed here, direct pediatric liquid-biopsy evidence is currently strongest for circulating miRNAs. This includes serum or plasma studies in juvenile pilocytic astrocytoma (Bookland et al. 2018), pediatric astrocytoma (López-Aguilar et al. 2017), DIPG (Iannó et al. 2022), pediatric glioma (Rogachevsky et al. 2026), intracranial malignant germ-cell tumors (Murray et al. 2020), ETMR (Madlener et al. 2023), and medulloblastoma (Xue et al. 2022). Direct pediatric evidence for other circulating RNA classes is considerably more limited; notably, Lee et al. identified medulloblastoma-associated circRNAs in CSF, whereas circulating piRNAs and tRNA-derived fragments have not yet been clinically validated in dedicated pediatric brain-tumor cohorts. In contrast, several studies included in this review derive from adult or non-pediatric glioma populations, including CSF miRNAs in glioblastoma and brain metastases (Teplyuk et al. 2012), plasma miRNA signatures in primary and recurrent glioblastoma (Bustos et al. 2022), extracellular-vesicle-associated piRNAs in glioma (Hallal et al. 2020), and circulating circRNAs in glioblastoma (Chen 2020). Huang et al. (2025) represents a further distinct category, as its glioma cohort was mixed-age but predominantly adult (age range 11–72 years; mean age 44 years), precluding pediatric-specific conclusions. Other observations, including those for tRF-19-R118LOJX/tRF-19-6SM83OJX and EV-associated circATP8B4, derive from genomic datasets and/or experimental glioma models rather than patient liquid biopsies (Wei et al. 2023; Zhao et al. 2019). Adult-derived and preclinical findings nevertheless remain relevant to pediatric research when interpreted as hypothesis-generating rather than clinically validated evidence. They demonstrate the analytical feasibility of detecting specific RNA classes in plasma, serum, CSF, or extracellular vesicles, identify candidate RNA-regulated pathways and molecular cargo for prioritization in pediatric models, and may inform assay design and biofluid selection. However, adult-derived signatures, effect sizes, and diagnostic thresholds should not be directly extrapolated to children and require independent validation in prospectively collected, molecularly defined pediatric cohorts. Table 1 therefore distinguishes direct pediatric clinical evidence from adult/non-pediatric, mixed-age, and preclinical evidence.

### MicroRNAs

MiRNAs are small non-coding RNA molecules approximately 22 nucleotides in length, primarily involved in the regulation of gene expression through gene silencing. miRNAs guide Argonaute proteins to the 3’ untranslated region (UTR) of target mRNAs, inhibiting translation and promoting mRNA degradation. Notably, a single miRNA can regulate multiple genes. As key epigenetic regulators, miRNAs play critical roles in both homeostatic and pathological cellular processes (Seo et al. 2024). In particular, miRNA dysregulation is deeply implicated in tumorigenesis and cancer progression, either by targeting oncogenes or tumor suppressors (Peng, et al. 2020). Furthermore, evidence suggests that under specific conditions, certain miRNAs can activate the translation of their target mRNAs. This occurs through interactions with miRNA-ribonucleoproteins (miRNPs) and RNA-binding proteins (RBPs) (Valinezhad Orang et al. 2014; Oliveto et al. 2017). Since 2008, increasing evidence has shown that endogenous circulating miRNAs (ct-miRNAs) are present in various biological fluids, including blood (serum and plasma), saliva, urine, CSF, and tears. These ct-miRNAs originate from direct cellular secretion, cell death, or trauma (Pozniak et al. 2022). Remarkably, they exhibit high stability against endogenous RNase activity (Mitchell et al. 2008), as they are protected by encapsulation in extracellular vesicles, by association with Ago2-containing ribonucleoprotein complexes, or by binding to high-density lipoproteins (Arroyo et al. 2011; Turchinovich et al. 2011; Vickers et al. 2011). Due to their exceptional stability, ct-miRNAs have emerged as promising biomarkers for diagnostic and prognostic applications even in biofluids.

Bookland et al. were pioneers in exploring the diagnostic potential of ct-miRNAs in pediatric brain tumors. Their study identified a signature of four ct-miRNAs (miR-21, miR-15 b, miR-23 a, and miR-146 b) in patients with juvenile pilocytic astrocytoma. Remarkably, the expression levels of these ct-miRNAs demonstrated the ability to predict both tumor nodular size and treatment response, achieving a sensitivity of 86% and a specificity of 100%.

A three-ct-miRNA serum signature (miR-130 a, miR-145, and miR-335) was shown to be a specific biomarker, with miR-130 upregulated and miR-145/335 downregulated in pediatric patients with astrocytoma compared with healthy controls (López-Aguilar et al. 2017).

Evidence from adult cohorts may nevertheless provide methodological and biological insights that can guide subsequent investigation in pediatric tumors. For example, in 2022, Bustos et al. observed differential expression of 193 ct-miRNAs in the plasma of patients with primary glioblastoma compared with healthy controls, and reported that miR-3180-3p and miR-5739 have potential utility for the diagnosis of GBM.

In addition to clinical studies, pediatric-derived preclinical models have provided mechanistic evidence supporting selected circulating RNA candidates. For example, distinct deregulation patterns were identified by analyzing exosomes derived from pediatric glioma stem cells compared with those from normal neural stem cells in vitro*.* Notably, miR-1290 and miR-1246 emerged as particularly significant, given their established association with “stemness” and invasive behavior in other cancers (Tűzesi et al. 2017).

In 2022, Iannó et al. identified a circulating 13-miRNA prognostic signature associated with disease progression in pediatric patients with diffuse intrinsic pontine glioma (DIPG). Serum samples from 47 patients enrolled in clinical trials combining nimotuzumab, vinorelbine, and radiotherapy were analyzed using a high-throughput miRNA profiling platform. The signature was developed in a training cohort (n = 23) and subsequently evaluated in a separate validation cohort (n = 24). In the validation cohort, the signature significantly stratified patients according to progression-free survival (PFS; HR = 5.51, 95% CI: 2.03–14.9; log-rank p = 0.00026). Importantly, the original study also reported quantitative measures of prognostic discrimination, including an area under the ROC curve (AUC) of 0.96 (95% CI: 0.888–1.000) at 8.5 months, with 88.9% sensitivity and 81.8% specificity. The Brier score at the same time point was 0.126, compared with 0.270 for the reference model without risk stratification. The signature was also associated with overall survival (HR = 4.12, 95% CI: 1.57–10.8; log-rank p = 0.0021). Although these findings support the potential prognostic value of circulating miRNAs in DIPG, the single-center cohorts and absence of prospective multicenter validation warrant further research (Iannó et al. 2022). Recently, Rogachevsky et al. (2026) evaluated a five-miRNA signature in plasma samples from 68 pediatric glioma patients and 12 healthy controls using real-time PCR. They demonstrated that all five circulating miRNAs were significantly upregulated in pediatric glioma patients compared with healthy controls and were significantly associated with glioma, with differential expression observed among different glioma subtypes. These findings suggest that the five-miRNA signature may represent a potential diagnostic tool for distinguishing pediatric glioma patients from healthy controls, as well as for discriminating between different glioma subgroups. From a mechanistic perspective, miR-182-5p showed the strongest association with glioma and promoted the migratory and invasive capacities of pediatric diffuse high-grade glioma 4 (pHGG-4) cells in vitro.

Among CNS tumors, germ-cell tumors (GCTs), a heterogeneous group of CNS and extra-CNS tumors thatalso occur in childhood, are characterized by epigenomic dysregulation (e.g., of miRNAs) (Almstrup et al. 2020). The currently used biomarkers alpha-fetoprotein (AFP) and human chorionic gonadotropin (HCG) demonstrate limited sensitivity and specificity in diagnosing malignant GCTs (Murray et al. 2020). Most intracranial malignant GCTs do not exhibit elevated AFP or HCG levels, necessitating biopsies for definitive diagnosis. However, intracranial GCTs are often located in surgically challenging areas, making biopsies risky procedures. Circulating miRNAs from the miR-371~373 and miR-302/367 clusters are consistently overexpressed in malignant GCTs, with miR-371 a-3p showing significantly elevated levels in serum and CSF at diagnosis (Murray et al. 2020), proving their applicability in distinguishing intracranial malignant GCTs from other tumor types (serum: miR-371 a-3p (p = 0.0159), miR-372-3p (p = 0.0095), miR-367 (p = 0.0190), miR-302 a (p = 0.0381), miR-302d-3p (p = 0.0159); CSF: miR-371 a-3p (p = 0.0286), miR-372-3p (p = 0.0028), miR-367-3p (p = 0.0167), miR-302d-3p (p = 0.0061) (Schönberger et al. 2023). However, these studies did not establish validated diagnostic thresholds or report robust estimates of clinical sensitivity and specificity for intracranial disease. Larger prospective cohorts are therefore required to determine diagnostic accuracy and clinical applicability.

In a liquid-biopsy study of pediatric patients with embryonal tumor with multilayered rosettes (ETMR), Madlener et al. (2023) proposed miR-517 a as a diagnostic markerfor ETMR, evaluating the expression of circulating miRNAs (6 CSF, 3 plasma, and 2 serum samples). Using a targeted NanoString approach, they observed that the relative expression of miR-517 a was significantly higher in ETMR tumor tissues. They then evaluated miRNA levels in serum and plasma by RT-PCR, observing the same pattern. Moreover, the ROC curve of miR-517 a in blood samples showed an AUC of ≃1. Finally, they proposed miR-517 a as a biomarker for disease monitoring, because its levels correlate with tumor volume.

Another descriptive study comparing pediatric patients with glioma and healthy controls found differential expression of 16 miRNAs by RT-PCR, but only in five patients, which limits its significance (Murray et al. 2020).

In addition to circulating miRNAs, exosomal miRNAs are alsorelevant in pediatric CNS tumors. For example, in 2021, Ping Xue et al. found that miR-101-3p and miR-423-5p levels in plasma exosomes from medulloblastoma patients were significantly higher than in healthy controls. In vitro functional analyses showed that miR-101-3p and miR-423-5p significantly inhibited tumor-cell proliferation, migration, colony formation, and invasiveness, and promoted apoptosis. They act as tumor suppressors by directly targeting the FOXP4 gene, a promoter of tumorigenesis. Furthermore, miR-101-3p targeted EZH2. In a murine model, they showed that overexpression of the two miRNAs inhibited tumorigenesis.

A study of ct-miRNAs in CSF revealed that miR-10 b and miR-21 levels were significantly increased in patients with glioblastoma and with brain metastases of lung and breast cancer, compared with patients with tumors in remission or non-neoplasticconditions (Teplyuk et al. 2012).

### PIWI-interacting RNAs (piRNAs)

PIWI-interacting RNAs (piRNAs) are small non-coding RNAs, typically 24–31 nucleotides in length, that associate with PIWI-family proteins and are best characterized for their role in transposable-element silencing and maintenance of genome stability. Although alterations in the PIWI/piRNA pathway have been described in several cancers, the evidence in pediatric brain tumors remains extremely limited. Importantly, our updated literature search did not identify dedicated pediatric medulloblastoma, ependymoma, diffuse midline glioma (DMG/DIPG), or pediatric high-grade glioma cohorts in which circulating piRNAs were clinically validated as diagnostic, prognostic, or monitoring biomarkers.

Available clinical evidence derives predominantly from adult or mixed-age glioma populations. Hallal et al. (2020) profiled small RNAs in extracellular vesicles (EVs) obtained from adult glioma patients and identified several differentially expressed piRNA-annotated sequences, including piR_016658, piR_016659, piR_020829, and piR_020490. Although serum-EV piRNA profiles distinguished glioma patients from healthy controls, they did not discriminate glioblastoma from grade II–III astrocytoma, indicating that their clinical specificity remains uncertain. More recently, Huang et al. (2025) performed comprehensive cell-free RNA profiling in CSF and plasma and identified piR-28347 among glioma-associated CSF features. However, this study included only 18 glioma patients spanning 11–72 years of age (mean, 44 years) and did not perform a pediatric-specific analysis. These findings should therefore be considered adult-derived or mixed-age proof-of-concept evidence rather than direct evidence for pediatric brain tumors.

Mechanistic observations may nevertheless identify pathways worth investigating in pediatric disease. PIWIL1 is highly expressed in adult glioblastoma stem-like cells and is required for their self-renewal and tumorigenicity (Huang et al. 2021), while piR-8041 has shown tumor-suppressive activity in experimental glioblastoma models (Jacobs et al. 2018). Such findings provide biological hypotheses but cannot currently be extrapolated to pediatric brain tumors or interpreted as evidence for circulating piRNA biomarkers. Moreover, particular caution is required when assigning small-RNA sequencing reads to piRNAs. Several sequences annotated as piRNAs in commonly used databases have subsequently been shown to correspond to fragments of tRNAs, rRNAs, YRNAs, snoRNAs, or other non-coding RNAs rather than bona fide PIWI-associated RNAs (Tosar et al. 2018). Future studies in molecularly defined pediatric brain tumors should therefore combine stringent small-RNA annotation with orthogonal validation and, where possible, evidence of PIWI association before candidate circulating piRNAs are considered for clinical development.

### tRNA-derived small RNAs

Transfer RNAs can generate regulated small RNA species collectively referred to as tRNA-derived small RNAs (tsRNAs), including tRNA-derived fragments (tRFs) and tRNA halves. These molecules participate in the regulation of translation, stress responses, gene expression, and intercellular signaling and have emerged as candidate biomarkers in several adult malignancies. However, direct clinical evidence for circulating tsRNAs in pediatric brain tumors is currently lacking, and no dedicated pediatric medulloblastoma, ependymoma, DMG/DIPG, or pediatric glioma cohort validating a circulating tRF/tsRNA signature was identified in our updated literature search.

Current evidence in glioma is derived primarily from adult tumor datasets, experimental models, or predominantly adult clinical cohorts. Wei et al. (2023) identified tRF-19-R118LOJX and tRF-19-6SM83OJX through analysis of TCGA LGG/GBM data and subsequently investigated them in human glioma cell lines. Both tRFs suppressed glioma-cell proliferation, migration, and vasculogenic mimicry, with tRF-19-R118LOJX acting at least partly through negative regulation of S100A11. These findings provide mechanistic evidence and identify potentially relevant pathways but do not constitute liquid-biopsy or pediatric clinical validation. Huang et al. (2025) subsequently demonstrated that tsRNAs constitute a substantial component of the CSF cell-free transcriptome and identified several diagnostically or prognostically associated tRFs, including tRF-32-FZ9KR959NRZZP and plasma tRF-21-RZ81JJ6R0/tRF-20-RZ81JJ6R. Nevertheless, because the glioma cohort was mixed-age and predominantly adult and pediatric-specific analyses were not reported, these candidates require independent validation in children.

### From established miRNA evidence to emerging piRNA and tsRNA biomarkers

The lack of pediatric piRNA and tsRNA studies contrasts with the broader and more mature pediatric evidence for circulating miRNAs. Recent pediatric investigations have extended miRNA profiling to multiple tumor entities and biofluids. Bookland et al. (2020) analyzed matched serum and tumor samples from children with juvenile pilocytic astrocytoma, medulloblastoma, and ependymoma; Coyle et al. (2022, conference abstract) profiled CSF and plasma from 54 children with different brain-tumor histologies and identified miRNA patterns associated with grade, dissemination, and survival; and Chapman et al. (2024; conference abstract) identified candidate CSF miRNAs for measurable residual disease detection in pediatric ependymoma. In medulloblastoma, prospective serial CSF collection within the Head Start 4 trial enabled Bell et al. (2022, conference abstract) to perform exosomal small-RNA sequencing in 27 pediatric patients and 11 non-tumor controls. Approximately 9,500 exosomal non-coding RNAs were detected, with marked differences at diagnosis and partial normalization after induction chemotherapy. Although this study demonstrates the feasibility of broad small-RNA profiling from only 1–2 mL of pediatric CSF, the reported analysis did not identify or validate piRNA- or tsRNA-specific biomarkers.

Several factors may explain this imbalance in the literature. miRNAs have benefited from a substantially longer period of biological and methodological investigation, and circulating miRNAs have been studied as cancer biomarkers since the seminal reports demonstrating their stability and detectability in serum and plasma in 2008 (Mitchell et al. 2008; Chen et al. 2008). Over time, this has supported the development of relatively mature nomenclature systems, reference databases, profiling platforms, and targeted validation assays. By contrast, mammalian piRNAs were identified later and their investigation in somatic tissues, cancers, and extracellular biofluids has developed more recently. Importantly, piRNA annotation remains challenging. Several sequences reported as piRNAs in somatic tissues have been shown to overlap other RNA fragments, raising concerns about misclassification and indicating that sequence detection alone may not be sufficient to establish PIWI association (Tosar et al. 2018). Current piRNA resources also remain affected by inconsistent nomenclature and incomplete or heterogeneous datasets, limiting cross-study comparison and reproducibility (Guo et al. 2024). tsRNAs face related but distinct challenges. They comprise a heterogeneous group of tRNA-derived fragments generated through multiple cleavage mechanisms, and their nomenclature is still not fully standardized. Moreover, extensive chemical modifications of parental tRNAs can interfere with reverse transcription and adapter ligation, generating substantial sequence-dependent biases and requiring specialized library-preparation and analytical methods that have only recently become widely available (Cabrelle et al. 2024; Padhiar et al. 2024). These technical and annotation limitations are further amplified in pediatric neuro-oncology by the rarity and molecular heterogeneity of individual tumor entities, small patient cohorts, limited availability of age-matched controls, and restricted blood and CSF volumes (Tripathy et al. 2023).

Thus, current evidence on small RNAs in liquid biopsies from pediatric patients remains predominantly miRNA-based, while piRNAs and tsRNAs represent promising but still largely unexplored classes requiring dedicated prospective pediatric investigation.

## Circular RNAs as emerging liquid-biopsy biomarkers

Circular RNAs (circRNAs) are covalently closed RNA molecules generated predominantly through back-splicing (Chen et al. 2023). Although they are not strictly classified as small RNAs, as many exceed 200 nucleotides in length, they are often analyzed alongside circulating small RNAs in liquid-biopsy studies and are emerging as promising biomarkers. Importantly, conventional small-RNA sequencing should not be regarded as a standard approach for circRNA detection or quantification, as its library-preparation procedures typically enrich short RNA fragments (approximately 18–30 nucleotides), thereby excluding most circRNAs. Reliable circRNA profiling generally requires dedicated RNA-sequencing strategies, appropriate library construction and size selection, and bioinformatic identification of back-splice junctions. Their closed structure confers resistance to exonuclease-mediated degradation, increasing their stability in biofluids. CircRNAs can regulate gene expression by interacting with miRNAs and RNA-binding proteins, acting as molecular scaffolds, and, in selected cases, encoding peptides (Chen et al. 2023; Kristensen et al. 2019; Xiao et al., 2020). Their incorporation into extracellular vesicles may also facilitate intercellular communication (Miao and Zhang 2023).

Liquid-biopsy evidence for circulating circRNAs in pediatric brain tumors is beginning to emerge, primarily from CSF, which is particularly informative given its direct contact with CNS tumors. Lee et al. (2022) profiled CSF from 40 children with medulloblastoma and 11 controls, identifying circ_463 and circ_273 as candidate biomarkers that distinguished patients from controls. By comparison, circRNA liquid biopsy is considerably more advanced in adult brain tumors: a plasma circFOXO3/circ_0029426/circ-SHPRH signature distinguished glioblastoma from controls with an AUC of 0.980 (Chen 2020), and a serum-exosomal circRNA panel was proposed for detecting high-grade astrocytoma (Li et al. 2022). These findings confirm that circRNAs are detectable and informative in blood and CSF, supporting their candidacy as pediatric biomarkers.

## Technical and pre-analytical challenges of circulating small-RNA detection in blood and CSF

### Choice of biofluid: plasma, serum, and CSF

The choice of biofluid represents a major source of between-study variability and should be defined according to the intended clinical application. Plasma and serum cannot be considered analytically interchangeable. During serum preparation, clot formation and platelet activation can modify the extracellular RNA pool, whereas plasma can retain variable numbers of platelets, leukocytes, cell fragments, and platelet-derived extracellular vesicles depending on the collection and centrifugation procedure. Several studies have demonstrated substantial differences between matched serum and plasma miRNA profiles, emphasizing that biomarker thresholds generated in one matrix should not be directly transferred to the other (Wang et al. 2012; Zendjabil 2024). Consequently, prospective biomarker studies should use a single predefined matrix throughout discovery and validation rather than pooling or directly comparing serum- and plasma-derived measurements.

For circulating cell-free RNA analyses, platelet-depleted plasma is attractive because it reduces the contribution of residual cellular material. However, serum remains a valid matrix when collection, clotting time, processing, and storage are rigorously standardized. The optimal choice may also depend on the molecular fraction under investigation. Studies specifically targeting EV-associated small RNAs require additional control of EV separation and co-isolated lipoproteins and should follow current extracellular-vesicle reporting recommendations, including MISEV2023 and MIBlood-EV (Zhang et al. 2024; Lucien et al. 2023).

CSF is biologically closer to CNS tumors and may provide a more favorable tumor-derived signal-to-background ratio, but its use in pediatric patients is constrained by invasiveness, low available volumes, and the frequent requirement for sedation during lumbar puncture. Moreover, CSF collection site (lumbar, ventricular, or intraoperative), traumatic blood contamination, initial volume, and processing conditions can substantially affect extracellular RNA and EV profiles. These variables therefore need to be explicitly reported. Importantly, the feasibility of molecular profiling from limited pediatric CSF volumes has already been demonstrated: in children with medulloblastoma enrolled in Head Start 4, exosomal small-RNA sequencing was successfully performed using only 1–2 mL of CSF and detected approximately 9,500 non-coding RNAs (Bell et al. 2022). This establishes technical feasibility but does not remove the need to define assay-specific limits of detection and quantification for individual biomarkers.

### Blood collection, hemolysis, processing, centrifugation, and storage

Pre-analytical standardization should begin at venipuncture. Collection tube type, anticoagulant, time from blood withdrawal to processing, transport conditions, centrifugation, and sample storage should be recorded for every specimen. Heparin is particularly problematic for PCR-based RNA assays because it inhibits reverse transcriptase and DNA polymerase; although heparinase treatment may partially alleviate this effect, EDTA-containing tubes are generally more compatible with downstream PCR workflows. Nevertheless, anticoagulants and specialized blood-preservation tubes can themselves influence extracellular RNA and EV profiles, and therefore tube type and manufacturer should remain constant within a study rather than being treated as interchangeable variables (Zendjabil 2024; Lucien et al. 2023).

Processing delay is another major confounder because prolonged contact with blood cells can result in cell activation, degradation, or release of intracellular RNAs. Whenever feasible, plasma should therefore be separated within approximately 2 h of venipuncture using a predefined time and temperature window. For total cell-free miRNA analysis, a two-step centrifugation approach is frequently used: an initial low-speed centrifugation separates plasma from blood cells, followed by a higher-speed centrifugation or filtration step to reduce residual platelets and cellular debris. Published protocols vary substantially in centrifugal force and duration; therefore, rather than assuming one universal protocol, studies should report centrifugal force in × g, duration, temperature, brake settings, transferred plasma volume, and number of centrifugation steps. Importantly, the processing workflow should be selected according to the intended analyte because high-speed processing that is appropriate for platelet-depleted cell-free RNA analysis may alter the EV fraction when EV-associated small RNAs are the specific endpoint.

Hemolysis is particularly critical because erythrocytes contain abundant miRNAs and even visually subtle hemolysis can substantially alter circulating miRNA measurements. Hemolysis should therefore be assessed objectively rather than only by visual inspection, for example through spectrophotometric measurement of free hemoglobin and/or miRNA-based indices such as the miR-451 a/miR-23 a relationship. Samples exceeding a prospectively defined hemolysis threshold should be excluded or analyzed separately. Platelet contamination should likewise be evaluated, particularly when platelet-enriched miRNAs are candidate biomarkers.

After processing, plasma or serum should be aliquoted before long-term storage, preferably at −80 °C, to minimize repeated freeze–thaw cycles. Although individual extracellular miRNAs can display considerable stability, the impact of freeze–thaw depends on matrix, carrier fraction, and analyte; therefore, the number of cycles should be minimized, matched across comparison groups, and explicitly reported rather than assuming universal stability.

Comparable principles apply to CSF. Prospective CSF studies should record the route and anatomical site of collection, starting volume, collection tube, time to processing, centrifugation conditions, storage duration, and number of freeze–thaw cycles. Samples should be centrifuged before storage when cell-free RNA or EV-associated RNA is the endpoint, and blood contamination from a traumatic tap should be quantified. The ISEV CSF Task Force specifically recommends reporting erythrocyte contamination and predefined exclusion thresholds, together with starting CSF volume and all processing and storage parameters (Sandau et al. 2024).

### RNA isolation, quality control, and normalization

RNA isolation represents an additional source of systematic variation. Low-concentration biofluids frequently contain RNA below the reliable range of conventional spectrophotometric assays, while extraction efficiency varies among phenol-based, column-based, and combined protocols. Commercial kits can preferentially recover different RNA species and size classes, and recovery may also depend on the initial biofluid volume. Therefore, the same RNA isolation procedure, input biofluid volume, elution volume, carrier strategy, and reagent lot should be maintained throughout discovery and validation whenever possible. Studies comparing several extraction methods have demonstrated kit-dependent differences in recovery of both endogenous circulating miRNAs and synthetic spike-ins, indicating that changing extraction protocols between cohorts can itself create apparent biological differences.

Exogenous synthetic RNAs added before extraction are useful for monitoring extraction efficiency and, when appropriate, additional spike-ins can be introduced before reverse transcription to evaluate RT efficiency. However, external spike-ins should be regarded primarily as technical process controls and should not automatically be used as biological normalizers. There is currently no universally stable endogenous circulating miRNA suitable for all biofluids, diseases, and experimental conditions. In particular, abundant blood-cell-derived miRNAs may be strongly influenced by hemolysis or residual platelets and should not be assumed to be stable reference genes.

For RT-qPCR studies, candidate endogenous reference miRNAs should therefore be empirically validated within the relevant matrix and cohort, preferably using more than one stable reference when relative quantification is required. For large profiling datasets, global or data-driven normalization may be preferable after appropriate quality filtering. Alternatively, targeted digital PCR can provide absolute target concentrations, which can be expressed relative to the original biofluid volume while retaining extraction and reverse-transcription spike-ins as process controls. Irrespective of the strategy, normalization procedures must be specified prospectively and retained unchanged in the validation cohort. RT-qPCR experiments should follow current MIQE recommendations, while digital-PCR assays should follow dMIQE guidelines, including reporting of assay efficiency or partition performance, dynamic range, limit of detection, limit of quantification, technical replicates, and handling of measurements below the quantifiable range (The dMIQE Group et al. 2020; Bustin et al. 2025).

### Analytical platforms: small-RNA sequencing, RT-qPCR, and digital PCR

No single analytical platform is optimal for all stages of circulating small RNA biomarker development. Small-RNA sequencing is particularly appropriate for discovery because it enables simultaneous interrogation of hundreds to thousands of known and potentially novel small RNAs and can provide sequence-level information on isomiRs and other RNA species. This breadth is particularly relevant to the present field because circulating miRNAs, piRNAs, and tRNA-derived fragments cannot be comprehensively investigated using a small predefined PCR panel. However, low RNA concentration in biofluids, stochastic sampling of rare molecules, library-preparation requirements, and sequence-dependent ligation biases can affect sensitivity and quantitative accuracy. Benchmark studies have shown substantial protocol-dependent bias in small-RNA sequencing, particularly at the adapter-ligation step, meaning that data obtained using different workflows for library preparation should not automatically be considered quantitatively equivalent (Hong et al. 2021; Androvic et al. 2022). It should be emphasized that conventional small-RNA sequencing is not an appropriate standard method for circRNA profiling, which requires dedicated library-preparation and bioinformatic strategies to detect back-splice junctions.

Small RNA library preparation protocols rely on size selection, which captures only molecules within a narrow length range (approximately 18–35 nt). Because circRNAs typically far exceed this size, they are essentially excluded during library preparation, making their profiling with this approach impractical. RT-qPCR remains highly suitable for targeted validation because it is sensitive, relatively inexpensive, widely available, and can quantify small panels from limited RNA inputs. For targets that are above the assay-specific lower limit of quantification, qPCR can provide high analytical precision. Its limitations include dependence on reverse-transcription efficiency, primer/probe specificity, amplification efficiency, reference-gene or calibrator selection, and the requirement for prior knowledge of the target. Cross-platform studies have demonstrated that apparently equivalent qPCR systems can differ considerably in the number of circulating miRNAs that they reliably quantify, reinforcing the need for platform-specific analytical validation rather than reliance on nominal Cq cutoffs (Hong et al. 2021).

Digital PCR, and particularly droplet digital PCR (ddPCR), is attractive for low-abundance candidate biomarkers because sample partitioning permits direct absolute quantification without an external calibration curve and provides high precision at low target copy number. In a direct comparison of RT-qPCR and ddPCR for circulating miRNA quantification, Campomenosi et al. (2016) evaluated miR-21, miR-126, and let-7 a in human serum samples. The two platforms demonstrated strong concordance (R^2^ = 0.963–0.984), while ddPCR showed comparable or improved precision, with a significantly lower coefficient of variation for let-7 a (p = 0.028). These findings support ddPCR as a reliable alternative for absolute circulating miRNA quantification, although its analytical advantages remain target- and assay-dependent. To compare ddPCR and real-time PCR for the quantification of low-abundance small RNAs, six miRNAs were analyzed in twofold dilution series spanning approximately 5 to 5,000 copies per reaction, with three technical replicates. ddPCR consistently displayed lower variation than real-time PCR, reducing the mean coefficient of variation by 37–86% for overall variation and by 48–72% for PCR-specific variation. However, ddPCR was not uniformly more sensitive than real-time PCR across the six miRNAs. Taken together, these findings suggest that the main advantage of ddPCR for low-copy miRNAs lies in its precision and reproducibility rather than in a lower detection limit (Hindson et al. 2013). Nevertheless, ddPCR is a targeted rather than discovery technology and has lower multiplexing capacity than sequencing, while threshold definition, partition number, Poisson sampling, reverse-transcription efficiency, and very high or very low template concentrations can still affect accuracy. Consequently, digital PCR should not be presented as intrinsically superior to qPCR or NGS; analytical sensitivity must be established for each individual assay using predefined LOD and LLOQ criteria.

For pediatric neuro-oncology, a pragmatic workflow is therefore to use small-RNA sequencing for discovery when sufficient material is available, followed by orthogonal validation of a restricted candidate panel using RT-qPCR or ddPCR in larger independent cohorts. For very limited CSF samples and a small number of predefined biomarkers, targeted PCR-based approaches may conserve material, whereas broad sequencing remains more informative during discovery. The demonstration that small-RNA sequencing is feasible from 1–2 mL of pediatric CSF suggests that these approaches are not mutually exclusive, but the choice should be determined by the biomarker-development phase and intended clinical use.

### Improving sensitivity for CNS-derived small RNAs in peripheral blood

The analytical challenge posed by peripheral blood is not solely one of assay sensitivity. CNS-derived molecules are biologically diluted after entering the systemic circulation and coexist with a much larger pool of small RNAs released by blood and peripheral tissues. Consequently, increasing analytical sensitivity alone cannot compensate for poor tumor specificity. Strategies should aim to improve both analytical sensitivity and the tumor-to-background ratio.

At the pre-analytical level, this includes minimizing hemolysis and platelet contamination and using a fixed platelet-depleted plasma workflow. At the analytical level, increasing the biofluid input within pediatric blood-volume constraints, concentrating extracellular RNA, performing replicate targeted measurements, and applying highly sensitive ddPCR assays can increase the probability of detecting low-copy targets. Targeted pre-amplification can also increase apparent sensitivity but should only be used after demonstrating that amplification efficiencies are comparable across targets and samples because pre-amplification can distort relative abundance.

Enrichment of EV-associated RNA represents another potential approach because vesicular encapsulation protects RNA from degradation and may enrich specific biological compartments. Immunoaffinity enrichment of tumor- or CNS-associated EV populations could theoretically improve signal-to-background ratios in blood. However, these strategies remain investigational: the specificity of several proposed neuronal EV surface markers remains debated, and EV separation procedures themselves can alter yield, purity, and RNA composition. Accordingly, EV-enrichment studies should follow MISEV2023/MIBlood-EV recommendations and demonstrate the specificity of the selected surface marker rather than assuming CNS origin.

Finally, candidate blood biomarkers should preferentially be selected not only for high analytical detectability but also for evidence of CNS/tumor enrichment. Whenever feasible, paired tumor, CSF, and blood analyses can distinguish genuinely tumor-associated molecules from systemic host responses. This distinction is particularly important in pediatric brain tumors, in which the small tumor-derived circulating RNA fraction can otherwise be obscured by hemolysis, immune-cell signals, platelet activation, corticosteroid exposure, radiotherapy, and other treatment-related effects.

## From mechanistic actionability to clinical validation of RNA biomarkers in pediatric brain tumors

Mechanistic studies suggest that the biological functions of non-coding RNAs may reveal therapeutically actionable vulnerabilities, although pathway actionability should be clearly distinguished from clinical validation of the corresponding circulating biomarker. In glioblastoma models, PIWIL1 is required for glioma stem-cell maintenance and tumorigenicity, and its silencing reduces tumor growth and prolongs survival in vivo (Huang et al. 2021). Similarly, restoration of the tumor-suppressive piR-8041 reduces glioma-cell proliferation and intracranial xenograft growth, suggesting that modulation of the PIWI/piRNA machinery may represent a potential therapeutic strategy; however, these observations derive from adult and preclinical models and have not been validated in pediatric brain tumors. The tRNA epitranscriptomic machinery may provide an even more direct therapeutic opportunity. PUS7-dependent tRNA pseudouridylation supports glioblastoma stem-cell tumorigenicity, while experimental small-molecule PUS7 inhibitors reduce pseudouridylation, suppress tumorigenesis, and prolong survival in preclinical models (Cui et al. 2021). Furthermore, tRF-19-R118LOJX and tRF-19-6SM83OJX suppress glioma-cell proliferation, migration, and vasculogenic mimicry, with tRF-19-R118LOJX acting, at least in part, through negative regulation of S100A11, thereby identifying a candidate molecular node connecting tRNA-derived RNAs with tumor vascular biology (Wei et al. 2023).

A more pediatric-specific example is provided by medulloblastoma, where the miR-520 h/CDK6 and miR-383-5p/FOXM1 axes and their expression in LB are regulated by circSKA3, thereby linking non-coding RNA circuitry to therapeutically tractable cell-cycle pathways. CircSKA3 depletion reduces medulloblastoma-cell proliferation, migration, invasion, and cell-cycle progression, whereas CDK6 represents the direct downstream target of miR-520 h (Liu et al. 2022). This observation is of translational interest because CDK4/6 inhibition has already entered pediatric and young-adult clinical development.

Although many clues suggest that circulating small RNAs could be important biomarkers in pediatric brain tumors, and their applicability in clinical practice deserves to be explored, their analysis is still not systematically performed in clinical trials.

For example, NCT04238819 evaluated abemaciclib combined with temozolomide, with or without irinotecan, in children with relapsed/refractory solid tumors, including CNS tumors, although enrollment was not based on circSKA3, miRNA expression, or any other circulating RNA biomarkers.

In medulloblastoma specifically, NCT06959979 is currently evaluating the response of patient-derived medulloblastoma organoids to palbociclib, ribociclib, and abemaciclib; importantly, the drugs are tested ex vivo rather than administered to enrolled patients. These examples illustrate how RNA-regulated pathways may identify pharmacologically actionable vulnerabilities, even when the RNA itself has not yet reached the stage of a predictive biomarker.

In contrast to these mechanistic advances, prospective clinical validation of circulating small-RNA biomarkers in pediatric neuro-oncology remains limited. The most directly relevant registered studies are summarized in Table 2. Importantly, they are predominantly observational biomarker studies, and none uses a circulating small-RNA result to assign treatment or select patients for a specific anticancer therapy.

**Table 2:** Registered clinical studies relevant to circulating RNA biomarkers in pediatric brain tumors. The studies include longitudinal evaluation of miRNAs in pediatric CNS tumors, a non-tumor pediatric control cohort, prospective molecular profiling in DIPG and other brainstem gliomas, and exploratory serum miRNA analyses. These studies remain predominantly observational or exploratory; none currently uses a circulating small-RNA result to prospectively assign or modify treatment or to function as a companion diagnostic. ClinicalTrials.gov records reviewed in October 2026. Status and enrollment are registry-reported, not evidence of published results; estimated and actual counts are distinguished where available. “Primary” and “secondary” refer to registered outcome measures

Study (sponsor, country)	Design, status, enrollment	Main enrollment criteria	Disease/population	Intervention/treatment context	Circulating RNA assessment (registered outcome)
**NCT01595126** (Ann & Robert H. Lurie Children’s Hospital of Chicago, USA)	Observational, prospective cohort; completed (12/2011–05/2021); 100 (registered)	Age 1–21 years; newly diagnosed, radiographically and/or histologically confirmed CNS tumor; no prior anticancer therapy except surgery (steroids permitted)	Pediatric CNS tumors	No registered intervention; standard-of-care treatment according to diagnosis	Primary outcome: presence of miRNAs and proteins in blood, CSF and urine from diagnosis through treatment (24 months), as potential markers of disease and treatment response
**NCT01556178** (Ann & Robert H. Lurie Children’s Hospital of Chicago, USA)	Observational; completed (07/2011–04/2012); 5 (actual)	Age 1–21 years; no CNS tumor; neurosurgery for hydrocephalus with routine blood/CSF sampling; CNS infection and demyelinating conditions excluded	Hydrocephalus; non-tumor pediatric control cohort	No registered intervention; no anticancer treatment	Primary outcome: miRNA levels in blood and CSF (2 years); secondary: survivin and other biological markers
**NCT01106794** (Children’s National Research Institute, USA)	Observational, prospective cohort; recruiting (start 04/2010; estimated primary completion 04/2025); 100 (estimated)	≤ 21 years; DIPG, other high-grade brainstem gliomas, or focal (WHO grade I/II) brainstem gliomas; gliomas outside the brainstem and other brainstem histologies (e.g., PNET) excluded	DIPG and brainstem gliomas	No registered intervention; specimens collected during therapy and/or at autopsy	Primary outcomes include genome-wide RNA expression profiling (Affymetrix) of tumor, normal brainstem tissue and CSF; serum and urine collected, but no RNA outcome registered for them; miRNAs/small RNAs not specified
**NCT05709522** (Aga Khan University, Pakistan)	Observational, prospective cohort; registry status recruiting, anticipated completion 11/2023 (last updated 02/2023); 80 anticipated (40 brain tumor, 40 blood cancer)	Age 5–21 years; newly diagnosed, untreated; recurrent or metastatic disease and pre-existing psychiatric/neurological illness excluded	Pediatric brain tumors; parallel blood-cancer cohort	Registered intervention: behavioral (neurocognitive and psychosocial assessment); no trial-assigned anticancer therapy	Secondary outcome: serum miR-21, miR-10 b and miR-210 by qRT-PCR (with GFAP, NSE, S100β), explored in relation to neurocognitive outcomes

Primary registry records: https://clinicaltrials.gov/study/NCT01595126; https://clinicaltrials.gov/study/NCT01556178; https://clinicaltrials.gov/study/NCT01106794; https://clinicaltrials.gov/study/NCT05709522; https://clinicaltrials.gov/study/NCT04238819; https://clinicaltrials.gov/study/NCT06959979

*Abbreviations*: *CNS* central nervous system, *CSF* cerebrospinal fluid, *DIPG* diffuse intrinsic pontine glioma, *miRNA* microRNA, *qRT-PCR* quantitative reverse-transcription polymerase chain reaction

Among these studies, NCT01595126 provides the most direct longitudinal evaluation of circulating miRNAs as biomarkers during treatment of pediatric CNS tumors, whereas NCT01556178 was designed primarily to provide a non-tumor reference cohort. NCT05709522 investigates selected serum miRNAs, but their role is exploratory and related primarily to neurocognitive outcomes rather than tumor diagnosis or therapeutic decision-making. NCT01106794 provides an important prospective framework for longitudinal molecular analysis of biofluids from patients with DIPG and other brainstem gliomas, but the registered protocol does not define a specific circulating small-RNA assay. Thus, among the studies identified, circulating small RNAs remain investigational biomarkers that may serve as potential companion diagnostics in the future.

This gap has important regulatory implications. Translation of a circulating small-RNA signature into clinical practice requires a clearly predefined context of use, rigorous control of pre-analytical variability, analytical validation of the measurement method, reproducible decision thresholds, and independent prospective demonstration of clinical performance and clinical utility. The FDA Biomarker Qualification Program specifically requires the biomarker to be supported within a well-defined context of use and considers analytical performance, sources of measurement variability, and the relationship between the biomarker, clinical outcome, and treatment where applicable. The recent FDA guidance on *Bioanalytical Method Validation for Biomarkers* further emphasizes validation of methods used to quantify biomarkers in clinical development. In Europe, Regulation (EU) 2017/746 on in vitro diagnostic medical devices (IVDR) requires demonstration of scientific validity, analytical performance, and clinical performance, requirements that are particularly demanding for novel analytes without established reference materials or reference measurement procedures. Finally, if a circulating small-RNA assay were used to identify patients expected to benefit from a specific treatment, it could meet the definition of a companion diagnostic under the IVDR, requiring notified-body conformity assessment and consultation with the relevant medicinal-product authority, including EMA where applicable.

## Conclusion

**Fig. 2:**
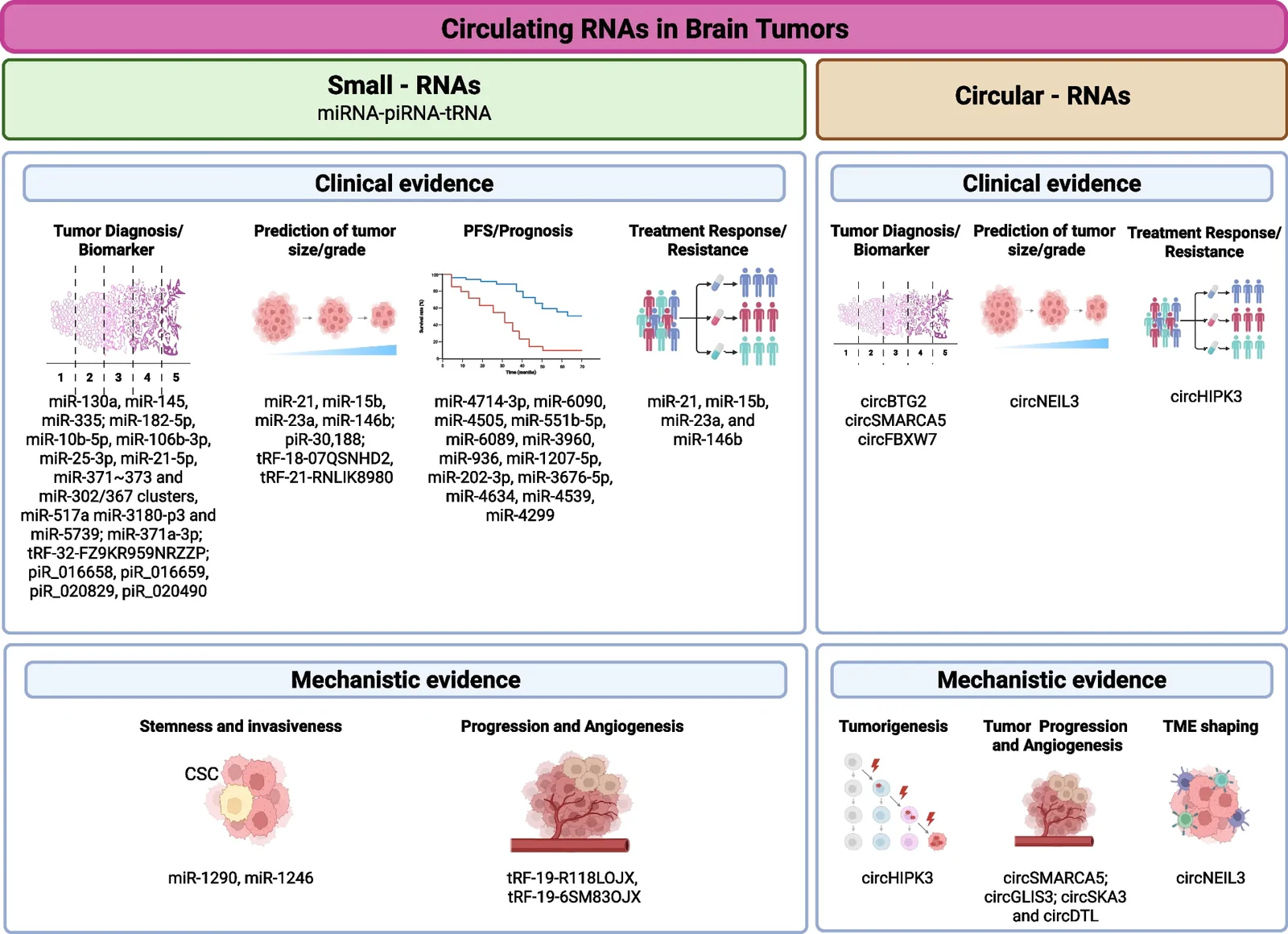
Overview of clinical and mechanistic evidence concerning small RNAs and circular RNAs in brain tumors. The figure distinguishes exploratory clinical biomarker findings from mechanistic observations derived from experimental models and tumor-associated molecular datasets. Clinical evidence includes studies conducted in pediatric and mixed-age populations, whereas mechanistic evidence primarily derives from tumor tissue analyses, cell-based experiments, and preclinical models. Evidence supporting circulating miRNAs is comparatively more developed, whereas circulating piRNAs, tsRNAs, and circRNAs remain largely exploratory, particularly in pediatric cohorts

A novel class of biomarkers is required to monitor the progression of the disease and the response to therapy in pediatric CNS tumors. Particularly because of tumor location, less invasive and lower-risk techniques are urgently needed. Circulating small RNAs represent a promising opportunity, especially in recent years, as their detection in both CSF and blood has garnered significant attention for diagnosis and disease monitoring (Fig. 2). Since circulating small RNAs are epigenetic regulators, their expression may vary rapidly; therefore, an important point of discussion is the need to reach a consensus on the timepoint of collection, sample type, and sample-processing technique. However, despite initial promise, most of these markers have not progressed beyond early discovery stages. A key challenge in translating these biomarkers into clinical practice is the inconsistency and lack of reproducibility between studies, often due to differences in sample preparation, analytical methods, or technologies used. These technical variations can introduce biases that distort results, affecting the measured expression of molecules in CSF samples. The diversity of available platforms can lead to conflicting conclusions, hindering the clinical application of these biomarkers. To move forward, it is crucial to address these technical issues and standardize procedures, ensuring the reliability of LB markers for diagnosing and monitoring CNS tumors (Lakkisto et al. 2023). Efforts to refine LB techniques are currently centered on enhancing the precision and accuracy of methods for detecting various tumor analytes in plasma, serum, and CSF samples. Biomarker development should use prospectively defined and standardized procedures for biofluid selection, collection, processing, hemolysis and platelet assessment, storage, RNA isolation, normalization, and analytical validation. The choice between small-RNA sequencing, RT-qPCR, and digital PCR should be driven by the intended phase of biomarker development and by available sample volume, with orthogonal validation across platforms whenever possible. These requirements are particularly important in pediatric neuro-oncology, where limited blood and CSF volumes amplify pre-analytical variability and where CNS-derived signals in peripheral blood may be strongly diluted by non-tumor RNA. Harmonized multicenter protocols and prospective validation in molecularly defined pediatric cohorts are therefore prerequisites for translating promising circulating small RNA signatures into clinically reproducible tests. Additionally, these approaches aim to broaden the range of molecular and pathological subtypes that can be identified, providing valuable support for future clinical decision-making. The integration of methylation-based molecular classification into clinical practice and the introduction of new tumor subtypes in the WHO 2021 classification of CNS tumors highlight the potential of LB as a non-invasive alternative for identifying these subtypes (Louis et al. 2021). To achieve widespread clinical application, these assays must undergo rigorous validation in additional prospective cohorts to confirm their reliability and utility. Looking ahead, non-invasive molecular profiling of CNS tumors has the potential to become part of routine clinical care, revolutionizing patient management by enabling safer, non-invasive diagnoses, minimizing surgical risks, and facilitating early detection of tumor emergence and progression. In conclusion, the pratical roadmap with the minimum requirements for clinical adoption of circulating smallRNAs is the following: i) Predefined context of use, the intended role of the biomarker (diagnosis, prognosis, response monitoring, or patient selection), the tumor type, the age group, and the biofluid (plasma, serum, or CSF); ii) Standardized pre-analytical workflow. Sample collection, processing time, hemolysis assessment, storage, and RNA extraction, with predefined acceptance criteria for sample quality; iii) Analytical validation of the assay. Establish sensitivity, specificity, precision, reproducibility, using an adequate method (e.g., RT-qPCR or ddPCR), with appropriate normalization strategies; iv) Predefined decision thresholds, RNA panel, algorithm, and cut-offs, and verify that they are reproducible; v) Prospective, multicenter clinical validation in pediatric cohorts. Test performance in independent, adequately powered cohorts with appropriate controls; vi) Demonstration of clinical utility, showing that the biomarker changes clinical management or improves outcomes.

## Supplementary Information

Supplementary Material 1

## Data Availability

No datasets were generated or analyzed during the current study.
