# Supplementary material for: Advancing liquid biopsies in pediatric brain tumors: unlocking the clinical utility of circulating cell-free small RNAs
Source: Adv Biotechnol (Singap). 2026 Nov 13;4(4):47. doi: 10.1007/s44307-026-00144-5 (PMC13768439; doi:10.1007/s44307-026-00144-5)
Supplement: Supplementary file 1 [file 44307_2026_144_MOESM1_ESM.docx]

**Table1.**

| **Functional category** | **RNA(s)** | **Tumor type(s)** | **Main biological role / pathway** | **Evidence supporting the classification** | **Translational relevance** | **References** |
| --- | --- | --- | --- | --- | --- | --- |
| **Tumor-intrinsic regulation** | **miR-21 / miR-21-5p** | Pediatric JPA; pediatric glioma; GBM | Oncogenic regulation of PTEN/PI3K-AKT, apoptosis and cell survival | Consistent association with tumor growth and malignant phenotype; circulating levels correlate with tumor burden in JPA | Potential diagnostic and tumor-burden biomarker | Bookland et al. 2018;  Rogachevsky et al. 2025;  Teplyuk NM et al. 2012 |
|  | **miR-15b** | Pediatric JPA | Regulation of cell-cycle and apoptotic pathways | Differential serum expression and correlation with tumor volume | Candidate tumor-burden biomarker | Bookland et al. 2018; |
|  | **miR-23a** | Pediatric JPA | Regulation of metabolic, survival and proliferation pathways | Differential serum expression and correlation with tumor volume | Candidate tumor-burden biomarker | Bookland et al. 2018; |
|  | **miR-146b** | Pediatric JPA; glioma | NF-κB and inflammatory signaling | Circulating association with JPA; established role in inflammatory regulation | Candidate biomarker linking tumor burden and inflammatory signaling | Bookland et al. 2018; |
|  | **miR-130a** | Pediatric astrocytoma | SDC4/EXT1/XYLT1-associated pathways | Differential expression in serum and tumor tissue | Candidate diagnostic biomarker | López-Aguilar JE et al. 2017 |
|  | **miR-145** | Pediatric astrocytoma | Differentiation and proliferation pathways | Reduced expression associated with astrocytoma | Potential tumor-suppressive biomarker | López-Aguilar JE et al. 2017 |
|  | **miR-335** | Pediatric astrocytoma | Migration/invasion and developmental pathways | Differential serum/tissue expression | Candidate biomarker; biological role requires further validation | López-Aguilar JE et al. 2017 |
|  | **miR-10b / miR-10b-5p** | Pediatric glioma; GBM; brain metastases | Regulation of migration/invasion pathways, including HOXD10/RhoC signaling | Circulating detection together with extensive functional evidence in glioma | Candidate disease-activity biomarker | Rogachevsky et al. 2025;  Teplyuk NM et al. 2012 |
|  | **miR-25-3p** | Pediatric glioma | Cell survival and proliferation; miR-106b~25 cluster | Differential plasma expression; supporting functional evidence in glioma | Candidate diagnostic biomarker | Rogachevsky et al. 2025; |
|  | **miR-106b-3p** | Pediatric glioma | Cell-cycle/proliferation regulation; miR-106b~25 cluster | Differential plasma expression and established oncogenic activity in cancer | Candidate diagnostic biomarker | Rogachevsky et al. 2025; |
|  | **miR-182-5p** | Pediatric high-grade glioma | Migration and invasion | Functional experiments show increased aggressiveness; inhibition reduces migration/invasion | Candidate marker of aggressive disease | Rogachevsky et al. 2025; |
|  | **miR-101-3p** | Medulloblastoma | **FOXP4 and EZH2** regulation | Exosomal transfer, in vitro functional assays and xenograft validation | Strong mechanistic and therapeutic relevance | Xue P et al. 2022 |
|  | **miR-423-5p** | Medulloblastoma | **FOXP4** regulation | Exosomal transfer, functional assays and in vivo validation | Strong mechanistic relevance | Xue P et al. 2022 |
|  | **tRF-19-R118LOJX** | Glioma | **S100A11** regulation | Functional studies demonstrate effects on proliferation, migration and vasculogenic mimicry | Potential prognostic/therapeutic relevance | Wei D., 2023 |
|  | **tRF-19-6SM83OJX** | Glioma | Regulation of malignant phenotype | Functional association with proliferation/migration | Potential biological biomarker | Wei D., 2023 |
| **Tumor–immune / TME communication** | **miR-1246** | Glioma | EV-mediated communication; immune/myeloid pathways | Tumor-derived EV studies demonstrate effects on recipient cells and MDSC-related pathways | Candidate regulator of immunosuppressive TME | Huang J. et al. 2025 |
|  | **miR-21** | Glioma/GBM | Inflammatory and immune-related signaling in addition to tumor-intrinsic survival pathways | Extensive glioma literature and CSF/plasma detection | Potential bridge between tumor burden and immune/TME state | Rogachevsky et al. 2025;  Teplyuk NM et al. 2012 |
|  | **miR-146b** | Pediatric JPA/glioma | NF-κB/inflammatory signaling | Established immune-regulatory function and circulating association | Candidate indicator of tumor-associated inflammation | Bookland et al. 2018; |
|  | **miR-101-3p** | Medulloblastoma | EV-mediated transfer to tumor cells | Direct demonstration of exosomal delivery and functional effects | Example of functional extracellular RNA communication | Xue P et al. 2022 |
|  | **miR-423-5p** | Medulloblastoma | EV-mediated transfer to tumor cells | Direct demonstration of exosomal delivery and functional effects | Example of functional extracellular RNA communication | Xue P et al. 2022 |
|  | **piR-016658, piR-016659, piR-020829, piR-020490** | GBM/LGG | EV-associated RNA cargo | Differential abundance in serum EVs | Candidate EV biomarkers; recipient-cell/TME effects not established | Hallal S, et al., 2020 |
| **Developmental / stemness programs** | **miR-371a-3p, miR-372-3p, miR-373-3p** | Intracranial germ-cell tumors | Germ-cell developmental and pluripotency-associated programs | Strong serum/CSF tumor association | Highly relevant diagnostic and treatment-monitoring biomarkers | Schönberger S et al. 2023 |
|  | **miR-302a/b/c/d, miR-367** | Intracranial germ-cell tumors | Pluripotency, stemness and developmental regulation | Expression associated with iGCT molecular phenotype | Candidate biomarkers reflecting tumor lineage/state | Schönberger S et al. 2023 |
|  | **miR-517a** | ETMR | C19MC-associated developmental program | Strong tumor-tissue and blood enrichment; circulating levels correlate with tumor volume | Particularly promising diagnostic and tumor-burden biomarker | Madlener S et al. 2023 |
|  | **miR-1246** | Glioma | Potential association with stem-like tumor phenotype | EV-associated and functional evidence | Candidate link between stemness, EV biology and TME | Huang J. et al. 2025 |
|  | **miR-1290** | Glioma | Regulation of glioma stem-like properties and survival | Functional/EV evidence | Potential biomarker of aggressive/stem-like disease | Huang J. et al. 2025 |
| **Biomarker-associated but biologically unresolved RNAs** | **miR-3180-3p, miR-5739** | GBM | Disease/recurrence-associated circulating signals | Plasma differential expression and diagnostic performance | Potential diagnostic/recurrence biomarkers; mechanisms unclear | Bustos Ma et al. 2022 |
|  | **miR-4714-3p, miR-6090, miR-4505, miR-551b-5p, miR-6089, miR-3960, miR-936, miR-1207-5p, miR-202-3p, miR-3676-5p, miR-4634, miR-4539, miR-4299** | DIPG | Composite prognostic miRNA signature | Prognostic association with PFS and OS; individual mechanisms poorly defined | Promising prognostic tool; individual contribution unresolved | Iannó MF et al. 2022 |
|  | **piR-28347** | Glioma | Component of CSF cfRNA signature | Signature-level diagnostic evidence | Candidate biomarker; individual function unknown | Huang J. et al. 2025 |
|  | **piR-2325** | Meningioma | Component of CSF cfRNA signature | Signature-level diagnostic evidence | Candidate biomarker; individual function unknown | Huang J. et al. 2025 |
|  | **tRF-21-RZ81JJ6R0, tRF-20-RZ81JJ6R** | Glioma | Recurrence/survival-associated cfRNAs | Individual prognostic associations in CSF/plasma | Potential prognostic biomarkers; mechanisms unknown | Huang J. et al. 2025 |
|  | **tRF-18-07QSNHD2, tRF-21-RNLIK8980** | Glioma | Disease-associated CSF cfRNAs | Differential expression in CSF | Candidate disease biomarkers; biological role unresolved | Huang J. et al. 2025 |
|  | **tRF-32-FZ9KR959NRZZP** | Glioma | Disease/recurrence-associated cfRNA | Clinical association | Candidate biomarker; biological function unresolved | Huang J. et al. 2025 |

**Table2.**

| **Functional category** | **RNA(s)** | **Tumor type(s)** | **Main biological role / pathway** | **Evidence supporting the classification** | **Translational relevance** | **References** |
| --- | --- | --- | --- | --- | --- | --- |
| **Tumor-intrinsic regulation** | **circFOXO3** | GBM | miRNA sponging involving **miR-138-5p/miR-432-5p → NFAT5** axis | Cellular and animal functional studies | Mechanistically relevant, but mainly tissue-based/adult evidence | Zhang S. et al. 2019 |
|  | **circ-SHPRH** | GBM | Encodes **SHPRH-146aa**, a tumor-suppressive peptide | Functional and animal studies demonstrate inhibition of GBM tumorigenicity | Strong biological relevance; not yet established as pediatric circulating biomarker | Chen S. et al. 2021 |
|  | **circ_0029426** | Glioma | Pro-tumorigenic regulation through miRNA-associated pathways | Functional studies in glioma models | Mechanistic evidence, but limited pediatric liquid-biopsy validation | Xiong H. et al. 2023 |
| **Tumor–immune / TME communication** | **circRNAs in serum exosomes**: circ_0003828, hsa_circ_0075828, hsa_circ_0002976 | High-grade astrocytoma | Stable EV-associated RNA cargo; potential intercellular signaling | Differential expression in serum exosomes; biological targets remain unresolved | Strong diagnostic potential; TME role remains to be demonstrated | Li P. et al. 2022 |
| **Biomarker-associated but biologically unresolved RNAs** | **circ_463, circ_273** | Medulloblastoma | Insufficiently characterized | Reported in the original candidate list, but mechanistic evidence requires verification | Potential biomarkers; insufficient biological evidence | - |
|  | **circRNA-ATP8B4** | Glioma | Insufficiently characterized | Primary functional evidence not sufficiently established | Candidate biomarker requiring validation | - |

Despite advances in the investigation of the biological functions of circulating RNAs, several remain of unknown significance despite their diagnostic/prognostic applications. While miRNAs field have been widely investigated, most piRNAs, tRFs identified through liquid-biopsy profiling remain biologically unresolved. Based on the available data, circulating RNAs in pediatric CNS tumors can be distinguished in several classes [ (i), (ii), (iii), (iv)] , not mutually exclusive, according to their exerted functions. (i) RNAs involved in tumor cells behaviour and survival: proliferation, apoptosis, migration and invasion; (ii) RNAs involved in TME regulation: tumor–immune system and tumor–microenvironment communication; (iii) RNAs associated with developmental and stemness programs; (iv) biological functions still insufficiently characterized to be pointed out. RNAs in this group are known to be disease associated biomarker. Table1. SmallRNAs
